# Predictive Model for Systemic Infection After Percutaneous Nephrolithotomy and Related Factors Analysis

**DOI:** 10.3389/fsurg.2021.696463

**Published:** 2021-07-23

**Authors:** Yiming Tang, Chi Zhang, Chengqiang Mo, Chengpeng Gui, Junhang Luo, Rongpei Wu

**Affiliations:** ^1^Department of Urology, The First Affiliated Hospital, Sun Yat-sen University, Guangzhou, China; ^2^Department of Urology, The Third Affiliated Hospital, Sun Yat-sen University, Guangzhou, China

**Keywords:** urolithiasis, prognostic factors, urosepsis, systemic inflammatory response syndrome, percutaneous nephrolithotomy

## Abstract

**Objectives:** To investigate the factors associated with systemic infection after percutaneous nephrolithotomy (PCNL) and establish a predictive model to provide theoretical basis for the prevention of systemic inflammatory response syndrome (SIRS) and urosepsis correlate to percutaneous nephrostomy.

**Methods:** Patients received PCNL between January 2016 and December 2020 were retrospectively enrolled. All patients were categorized into groups according to postoperative SIRS and urosepsis status. Single factor analysis and multivariate logistic regression analysis were performed to determine the predictive factors of SIRS and urosepsis after PCNL. The nomograms were generated using the predictors respectively and the discriminative ability of was assessed by analyses of receiver operating characteristic curves (ROC curves).

**Results:** A total of 758 PCNL patients were enrolled in this study, including 97 (12.8%) patients with SIRS and 42 (5.5%) patients with urosepsis. Multivariate logistic regression analysis suggested that there were 5 factors related to SIRS, followed by preoperative neutrophil to lymphocyte ratio (NLR) (odds ratio, OR = 1.721, 95% confidence interval, CI [1.116–2.653], *p* = 0.014), S.T.O.N.E. score (OR = 1.902, 95% CI [1.473–2.457], *p* < 0.001), female gender (OR = 2.545, 95% CI [1.563–4.144], *p* < 0.001), diabetes history (OR = 1.987, 95% CI [1.051–3.755], *p* = 0.035), positive urine culture (OR = 3.184, 95% CI [1.697–5.974], *p* < 0.001). And there were four factors related to urosepsis, followed by preoperative NLR (OR = 1.604, 95% CI [1.135–2.266], *p* = 0.007), S.T.O.N.E. score (OR = 1.455, 95% CI [1.064–1.988], *p* = 0.019), female gender (OR = 2.08, 95% CI [1.063–4.07], *p* = 0.032), positive urine culture (OR = 2.827, 95% CI [1.266–6.313], *p* = 0.011). A nomogram prediction model was established to calculate the cumulative probability of SIRS and urosepsis after PCNL and displayed favorable fitting by Hosmer–Lemeshow test (*p* = 0.953, *p* = 0.872). The area under the ROC curve was 0.784 (SIRS) and 0.772 (urosepsis) respectively.

**Conclusion:** Higher preoperative NLR, higher S.T.O.N.E. score, female gender, and positive urine culture are the most significant predictors of SIRS and urosepsis. Diabetes history is the predictor of SIRS. These data will help identify high-risk individuals and facilitate early detection of SIRS and urosepsis post-PCNL.

## Introduction

Since the first report of percutaneous nephrolithotomy (PCNL) in 1976, PCNL has gradually become the main method of treatment for upper urinary tract stones (1, 2). Although PCNL is a minimally invasive surgery, it is still invasive to a certain extent, and the corresponding intraoperative and postoperative complications are also more common than other endourological procedures, such as bleeding, extravasation, residual stones, systemic inflammatory response syndrome (SIRS) and even urosepsis (3, 4). Severe cases can be life-threatening due to the postoperative treatment is not promptly (5). Timely and effective treatment of patients in the first 6 h of sepsis, including effective antibacterial treatment and maintenance of circulation perfusion, can significantly reduce the lethal rate of urosepsis (6).

In clinical practice, the initial stage of sepsis often lacks typical clinical symptoms, which makes it difficult to identify the occurrence of sepsis at an early stage (4). The best opportunity for early treatment will be missed if it does not attract enough attention until develops into severe sepsis and occurs the corresponding clinical pathophysiological changes. It is difficult to obtain good results even if the treatment is intensified in the later stage (7). At present, blood bacterial culture is an important basis for diagnosing sepsis and guides the use of antibiotics (8). But it cannot give specific culture results and provide effective anti-infection treatment early due to the long culture period and low positive rate.

In this study, our objective was to establish an early clinical risk prediction model for post-PCNL SIRS and urosepsis by evaluating the correlation between preoperative and intraoperative indicators.

## Materials and Methods

From January 2016 to December 2020, 758 kidney stone patients undergone PCNL were recruited in the study. Urinary non-contrast computed tomography (CT) was used for the diagnosis of kidney stones. Patients were excluded from the analysis for the following situations: (a) concurrently combined with other surgical methods such as ureteroscopic lithotripsy; (b) patients with tumors, blood system and immune system diseases or cases of hyperthyroidism; (c) congenital malformations such as polycystic kidney and horseshoe kidney; (d) lack of preoperative CT scan image data of urinary system.

Patients' preoperative information was collected including: gender, age, body mass index (BMI), diabetes history, ureteral stenting, nephrostomy, peripheral blood white blood cell (WBC), lymphocytes (L), neutrophils (N), NLR, platelet (PLT), hemoglobin (HB), serum creatinine, uric acid, urine WBC, urine culture, urine nitrite, preoperative antibiotics application, S.T.O.N.E. score (9). Intraoperative information includes: operation time, intraoperative blood loss, number of percutaneous access. Postoperative information were uniformly measured at 6 am on the second day after surgery include: peripheral WBC, blood pressure, heart rate, oxygenation, respiratory rate, body temperature, Glasgow Coma Scale (GCS) score. SIRS was defined as the occurrence of any 2 or more of the following 4 criteria: leukocyte count <4,000 or >12,000 cells/ul, body temperature >38 or <36°C, heart rate >90 /min, respiratory rate >20/min or PaCO2 <32 mmHg (10). Urosepsis was defined as ≥2 criteria of the qSOFA (quick sepsis-related organ failure assessment) score: Respiratory rate ≥ 22/min; Altered mentation (GCS score <13); Systolic blood pressure ≤100 mmHg (11).

All patients were categorized into groups according to postoperative SIRS and urosepsis status. All patients undergoing PCNL were used antibiotics prophylactically during the operation and patients who did not develop SIRS or urosepsis postoperative will also use antibiotics until 48 h after PCNL surgery, while the duration of postoperative antibiotics used for patients who considered SIRS or urosepsis after PCNL depended on whether their infection was controlled or not. Chi-square test was used to compare categorical variables. Student's *t*-test was used to compare the means of continuous variables between groups. Variables that showed significant differences were included in a multivariate logistic regression analysis. A nomogram based on the multivariate logistic regression results was set up to predict postoperative SIRS and urosepsis. 1,000 bootstrap samples were used to generate a calibration curve to reduce over-fitting bias. Hosmer–Lemeshow test implied good calibration when the test is insignificant. The discriminative performance was assessed by area under the ROC curve. Statistical analyses were performed using the SPSS version 22.0 and R version 4.0.3. Two-tailed *p* < 0.05 were considered statistically significant.

## Results

From January 2016 to December 2020, a total of 758 patients were diagnosed with upper urinary calculi in the Department of Urology, The First Affiliated Hospital of Sun Yat-sen University and received PCNL treatment. Among them, 97 cases were considered SIRS, accounting for 12.8% of the total; 42 patients with urosepsis were diagnosed postoperatively, accounting for 5.5% of the total.

The results of *t* test or chi-square test on the selected 22 factors show that there are 9 factors related to SIRS. They are higher preoperative WBC (*p* = 0.002), higher preoperative N (*p* < 0.001), higher preoperative NLR (*p* < 0.001), higher S.T.O.N.E. score (*p* < 0.001), female gender (*p* < 0.001), diabetes history (*p* = 0.005), urine WBC ≥ 50 cells/mL (*p* < 0.001), positive urine nitrite (*p* < 0.001), positive urine culture (*p* < 0.001). The characteristics of patients who developed sepsis included higher preoperative N (*p* = 0.001), higher preoperative NLR (*p* < 0.001), higher S.T.O.N.E. score (*p* = 0.005), female gender (*p* = 0.01), positive urine nitrite (*p* < 0.001), positive urine culture (*p* < 0.001), all data are detailed in Table 1.

**Table 1:** Clinical characteristics and single factor analysis of risk factors in patients with SIRS and sepsis after PCNL.

**Variable**	**SIRS (*n* = 97)**	**Non-SIRS (*n* = 661)**	***p* value**	**Urosepsis (*n* = 42)**	**Non-urosepsis (*n* = 716)**	***p* value**
Age (year), mean (SD)	52.5 (11.9)	51.9 (12.7)	0.673	53.0 (14.0)	52.0(12.5)	0.601
BMI (kg/m^2^), mean(SD)	22.5 (3.0)	22.9 (2.9)	0.272	22.6 (2.8)	22.9(2.9)	0.662
Preoperative WBC (10^∧^9/L), mean (SD)	7.5 (2.6)	6.6 (1.6)	**0.002**	7.2 (2.2)	6.7(1.8)	0.072
Preoperative L (10^∧^9/L), mean (SD)	2.1 (0.9)	2.1 (0.7)	0.656	1.9 (0.9)	2.1(0.7)	0.123
Preoperative N (10^∧^9/L), mean (SD)	4.5 (1.7)	3.7 (1.2)	**<0.001**	4.5 (1.4)	3.8(1.3)	**0.001**
Preoperative NLR, mean (SD)	2.5 (1.0)	2.0 (0.9)	**<0.001**	2.6 (1.1)	2.0(0.9)	**<0.001**
Preoperative PLT (10^∧^9/L), mean (SD)	286.1 (86.3)	283.7 (75.0)	0.779	273.3 (78.2)	284.7(76.4)	0.350
Preoperative HB (g/L), mean (SD)	133.7 (17.2)	136.0 (15.9)	0.194	135.8 (13.1)	135.8(16.2)	0.998
Preoperative serum creatinine (umol/L), mean (SD)	106.0 (71.4)	107.6 (77.9)	0.857	98.1 (60.6)	107.9(77.9)	0.428
Preoperative uricacid (umol/L), mean (SD)	396.4 (114.5)	413.8 (104.5)	0.132	392.8 (117.6)	412.6(105.1)	0.238
Operation time (min), mean (SD)	125.8 (41.2)	118.7 (26.2)	0.102	123.8 (45.2)	119.3(27.4)	0.528
Intraoperative blood loss (ml), mean (SD)	39.7 (31.7)	39.5 (28.9)	0.932	38.9 (26.1)	39.5(29.5)	0.896
S.T.O.N.E. score, mean (SD)	9.0 (1.0)	8.4 (1.1)	**<0.001**	8.9 (1.0)	8.4(1.1)	**0.005**
Gender, *n* (%)						
Male	40 (41.2%)	411 (62.2%)		17 (40.5%)	434(60.6%)	
Female	57 (58.8%)	250 (37.8%)	**<0.001**	25 (59.5%)	282(39.4%)	**0.010**
Diabetes, *n* (%)						
Yes	21 (21.6%)	76 (11.5%)		9 (21.4%)	88(12.3%)	
No	76 (78.4%)	585 (88.5%)	**0.005**	33 (78.6%)	628(87.7%)	0.085
Previous DJ indwelled, *n* (%)						
Yes	3 (3.1%)	19 (2.9%)		1 (2.4%)	21(2.9%)	
No	94 (96.9%)	642 (97.1%)	1.000	41 (97.6%)	695(97.1%)	1.000
Previous nephrostomy, *n* (%)						
Yes	3 (3.1%)	42 (6.4%)		2 (4.8%)	43(6.0%)	
No	94 (96.9%)	619 (93.6%)	0.299	40 (95.2%)	673 (94.0%)	1.000
Urine WBC, *n* (%)						
≥50 cells/uL	28 (28.9%)	131 (19.8%)		13 (31.0%)	146 (20.4%)	
<50 cells/uL	69 (71.1%)	530 (80.2%)	**0.041**	29 (69.0%)	570 (79.6%)	0.102
Urine nitrite, *n* (%)						
Positive	31 (32.0%)	84 (12.7%)		15 (35.7%)	100 (14.0%)	
Negative	66 (68.0%)	577 (87.3%)	**<0.001**	27 (64.3%)	616 (86.0%)	**<0.001**
Urine culture, *n* (%)						
Positive	37 (38.1%)	98 (14.8%)		18 (42.9%)	117 (16.3%)	
Negative	60 (61.9%)	563 (85.2%)	**<0.001**	24 (57.1%)	599 (83.7%)	**<0.001**
Antibiotics before surgery, *n* (%)						
Positive	19 (19.6%)	117 (17.7%)		8 (19.0%)	128 (17.9%)	
Negative	78 (80.4%)	544 (82.3%)	0.651	34 (81.0%)	588 (82.1%)	0.848
Number of channels, *n* (%)						
≥2	12 (12.4%)	46 (7.0%)		5 (11.9%)	53 (7.4%)	
<2	85 (87.6%)	615 (93.0%)	0.061	35 (88.1%)	663 (92.6%)	0.286

*Bold values indicate statistically significant (p < 0.05)*.

*SIRS, systemic inflammatory response syndrome; PCNL, percutaneous nephrolithotomy; SD, standard deviation; BMI, body mass index; WBC, white blood cell; L, lymphocytes; N, neutrophils; NLR, neutrophil to lymphocyte ratio; PLT, platelet; HB, hemoglobin; DJ, double J tube*.

The single factors related to SIRS and urosepsis were subjected to multivariate logistic regression analysis. The results showed that there were five factors related to SIRS (Table 2), followed by preoperative NLR (OR = 1.721, 95% CI [1.116–2.653], *p* = 0.014), S.T.O.N.E. score (OR = 1.902, 95% CI [1.473–2.457], *p* < 0.001), female gender (OR = 2.545, 95% CI [1.563–4.144], *p* < 0.001), diabetes history (OR = 1.987, 95% CI [1.051–3.755], *p* = 0.035), positive urine culture (OR = 3.184, 95% CI [1.697–5.974], *p* < 0.001). There were four factors related to urosepsis (Table 3), followed by preoperative NLR (OR = 1.604, 95% CI [1.135–2.266], *p* = 0.007), S.T.O.N.E. score (OR = 1.455, 95% CI [1.064–1.988], *p* = 0.019), female gender (OR = 2.08, 95% CI [1.063–4.07], *p* = 0.032), positive urine culture (OR = 2.827, 95% CI [1.266–6.313], *p* = 0.011).

**Table 2:** Multivariable logistic regression analysis of predictors of SIRS after PCNL.

**Variable**	**B**	**SE**	**Wald**	**OR**	**95% CI**		***p* value**
Preoperative WBC	0.373	0.216	2.99	1.452	0.951	2.215	0.084
Preoperative N	−0.217	0.333	0.423	0.805	0.419	1.547	0.515
Preoperative NLR	0.543	0.221	6.046	1.721	1.116	2.653	**0.014**
S.T.O.N.E. score	0.643	0.131	24.227	1.902	1.473	2.457	**<0.001**
Female gender	0.934	0.249	14.093	2.545	1.563	4.144	**<0.001**
Diabetes	0.686	0.325	4.465	1.987	1.051	3.755	**0.035**
Urine WBC ≥ 50 cells/ul	−0.12	0.309	0.151	0.887	0.485	1.624	0.698
Positive urine nitrite	0.626	0.327	3.665	1.869	0.985	3.546	0.056
Positive urine culture	1.158	0.321	13.016	3.184	1.697	5.974	**<0.001**

*Bold values indicate statistically significant (p < 0.05)*.

*B, regression coefficient; SE, standard error; OR, Odds Risk*.

**Table 3:** Multivariable logistic regression analysis of predictors of urosepsis after PCNL.

**Variable**	**B**	**SE**	**Wald**	**OR**	**95% CI**		***p* value**
Preoperative N	0.194	0.124	2.448	1.214	0.952	1.547	0.118
Preoperative NLR	0.473	0.176	7.181	1.604	1.135	2.266	**0.007**
S.T.O.N.E. score	0.375	0.159	5.532	1.455	1.064	1.988	**0.019**
Female gender	0.732	0.342	4.577	2.08	1.063	4.07	**0.032**
Positive urine nitrite	0.623	0.427	2.132	1.865	0.808	4.305	0.144
Positive urine culture	1.039	0.41	6.432	2.827	1.266	6.313	**0.011**

*Bold values indicate statistically significant (p < 0.05)*.

*B, regression coefficient; SE, standard error; OR, Odds Risk*.

**Figure 1:**
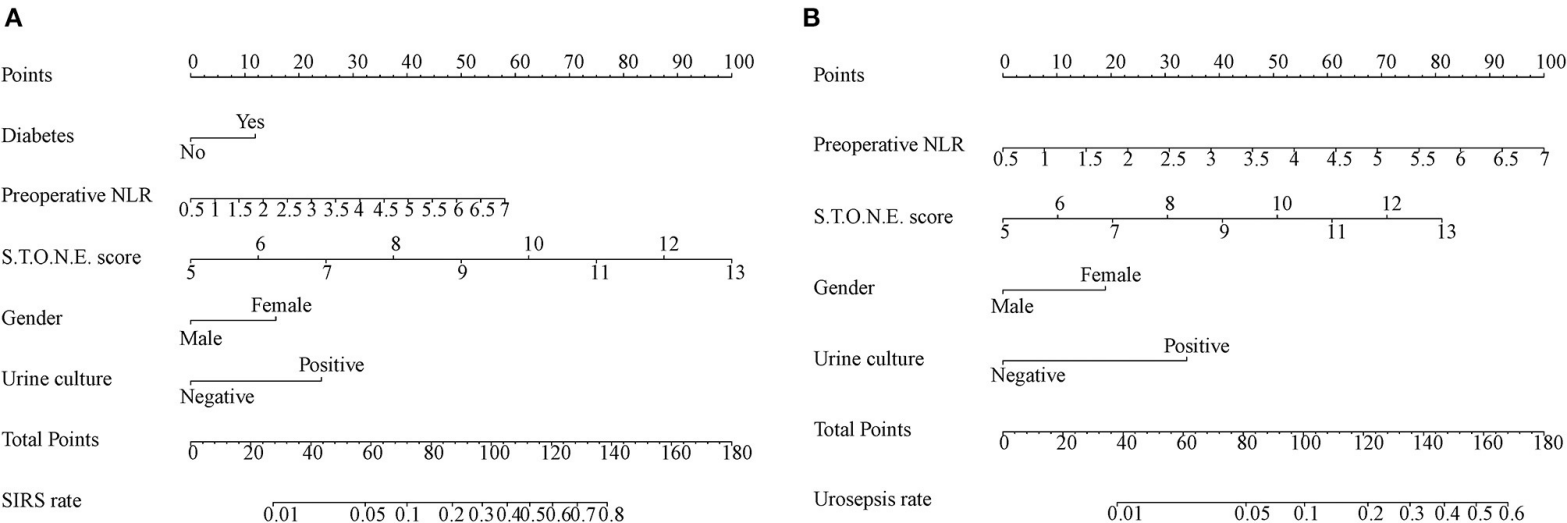
Nomogram developed for patients predicting post-operative SIRS **(A)** and urosepsis **(B)** NLR: neutrophil to lymphocyte ratio. Diabetes; Preoperative NLR; S.T.O.N.E. score; Gender; Urine culture are marked as points. The respective points can be added to calculate the incidence of SIRS and urosepsis.

**Figure 2:**
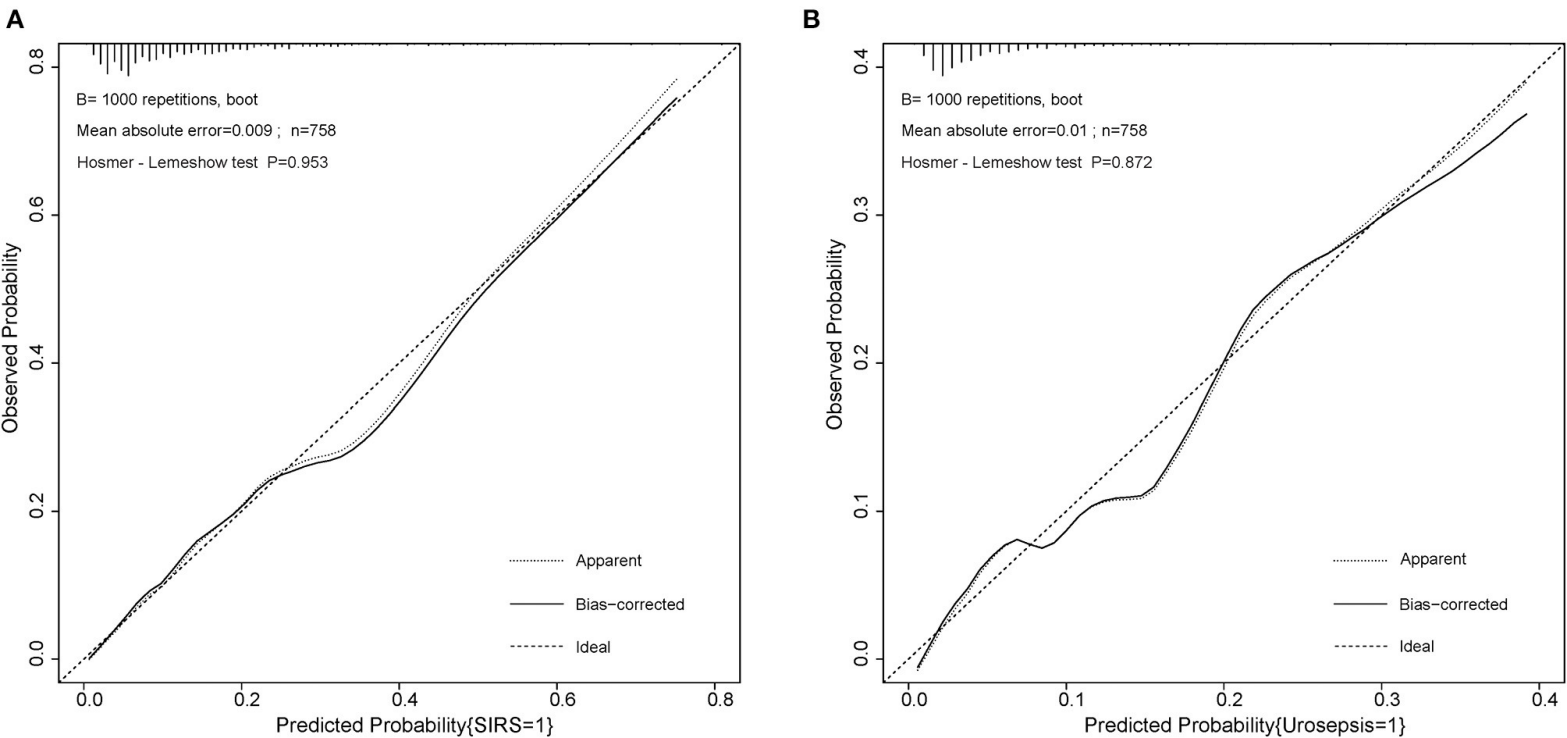
Calibration curve of the two nomogram for post-operative SIRS and urosepsis. **(A)** Hosmer–Lemeshow test with insignificant *p* value (0.953) indicates good fitting of SIRS model. **(B)** Hosmer–Lemeshow test with insignificant *p* value (0.872) also indicates good fitting of urosepsis model.

**Figure 3:**
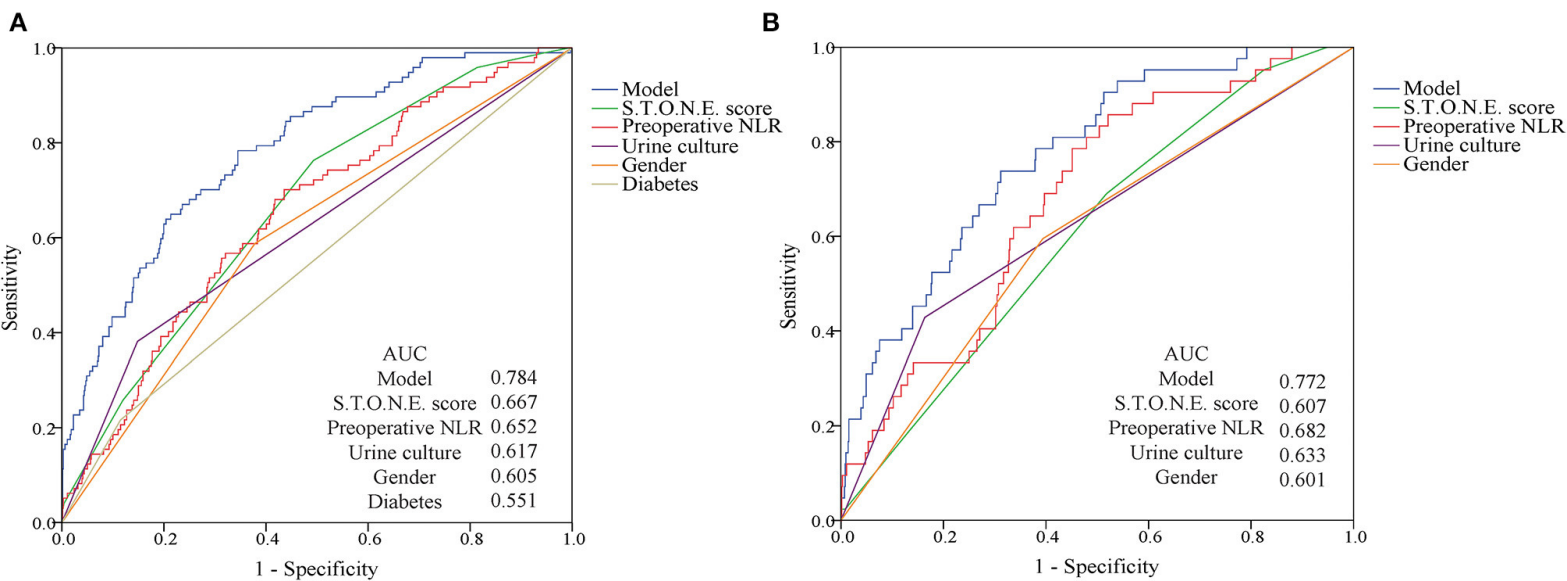
ROC curves for post-operative SIRS and urosepsis. **(A)** The AUC for the SIRS model is 0.784. **(B)** The AUC for the urosepsis model is 0.772. Both have favorable ability of discrimination.

On the basis of multi-factor analysis, a nomogram prediction model was established to calculate the cumulative probability of SIRS and urosepsis after PCNL (Figures 1A,B). The incidence of SIRS and urosepsis can be reflected by adding the points assigned to the five factors and four factors to obtain the total scale score. The calibration curve shows that the model fits well through the Hosmer–Lemeshow test, and there is no statistical significance (*p* = 0.953, *p* = 0.872) (Figures 2A,B). The ROC curve was drawn by SPSS software according to the predicted probability and actual postoperative SIRS occurrence (Area Under Curve, AUC was 0.784, 95% CI [0.736–0.832], Youden index = 0.439, sensitivity = 78.4%, specificity = 65.5%, *p* < 0.001) (Figure 3A). The same method obtains the ROC curve of the prediction model related to urosepsis (AUC = 0.772, 95% CI [0.705–0.839], Youden index = 0.427, sensitivity = 73.8%, specificity = 68.9%, *p* < 0.001) (Figure 3B).

In clinical work, we can add the scores corresponding to each independent risk factor of a patient on the nomogram to obtain the total score. Then the total score corresponds to the corresponding probability of infection and can be used to predict the occurrence of SIRS and urosepsis after surgery.

## Discussion

Compared with open and laparoscopic surgery, PCNL has the advantages of less trauma, high stone removal efficiency, mild postoperative pain, less bleeding, and quick recovery. It has now become a common surgical method for the treatment of upper urinary tract stones (12). However, compared with other minimally invasive stone surgery techniques, PCNL is still the most invasive surgical method, and systemic inflammatory infection is one of the most common complications after PCNL. If it is not paid attention in the early stage, there is no effective intervention. It is very likely to progress to SIRS and even urosepsis, which seriously endangers the life safety of patients [5]. Systemic inflammatory infection after PCNL is not uncommon, Previous studies have shown that the probability of SIRS after PCNL is between 7.7 and 31.1% (13, 14). The incidence of SIRS after PCNL is 12.8% (97/758) in our study, which is similar to previous reports. And the urosepsis rate reached 5.5% (42/758) in our research, which was consistent with previous studies (0.97–5.9%) (15, 16). In this study, we used logistic multivariate analysis to screen five factors related to postoperative SIRS: preoperative NLR (*P* = 0.014), S.T.O.N.E. score (*p* < 0.001), female gender (*p* < 0.001), diabetes history (*p* = 0.035) and positive urine culture (*p* < 0.001). Four factors related to postoperative urosepsis were screened: preoperative NLR (*p* = 0.007), S.T.O.N.E. score (*p* = 0.026), female gender (*p* = 0.033), positive urine culture (*p* = 0.01). We tried to early evaluate the possibility of postoperative SIRS and urosepsis with these factors.

According to our best knowledge of literatures, it is the first time to reveal S.T.O.N.E. score as an independent predictor of post-PCNL SIRS and urosepsis (OR = 1.908, *p* < 0.001; OR = 1.436, *p* = 0.026). The S.T.O.N.E. score consists of five stone characteristics: stone size (S), tract length (T), obstruction (O), number of involved calices (N), and essence or stone density (E). It was first proposed by Okhunov et al. to evaluate the complexity of kidney stones on the basis of kidney CT plain scan and had been used to evaluate the effect of percutaneous nephroscope stone removal and predict the length of surgery (9). Among them, the largest cross-sectional area of the stone reflects the load of the stone, the degree of obstruction and the number of involved renal calyxes reflect the condition of the renal pelvis, and the distance of the skin-renal passage and the density of the stone can reflect the length of operation (17). In this study, patients with higher S.T.O.N.E. scores are more likely to have systemic inflammatory infections after surgery. The main reason is that when the stone load is large, the distance between the skin and kidney channels is long, and the stone density is high, the operation time is correspondingly prolonged. When the diameter of the stone is >20 mm, the stone is likely to contain pathogens and endotoxins (18). When the stone is crushed, the pathogens and endotoxins in the stone will be released into the irrigation fluid and absorbed into the blood. In addition, when the urinary tract is obstructed, the pressure in the renal pelvis and calyces will increase correspondingly when the infective urine is not drained smoothly, which not only facilitates the reproduction and invasion of bacteria, but also increases the difficulty of antibacterial treatment (19). Therefore, for patients with high S.T.O.N.E. scores, adequate preoperative preparations should be made. Active anti-infective treatment during the perioperative period, reduction of intraoperative perfusion and, if necessary, staged surgery can help reduce the possibility of postoperative systemic inflammation and infection.

In this study, 57 of 97 patients considered for postoperative SIRS were female, accounting for 58.8%, and among patients with postoperative urosepsis, female accounted for 59.5% (25/42). Multivariate analysis indicated that female gender was one of the independent risk factors for postoperative SIRS and urosepsis. This is related to the physiological and anatomical characteristics of the urethra in female patients. Female urethra is short and the urethral opening is close to the vagina and anus, which is easy to be contaminated. When the local resistance is weakened, it is easy to cause urinary tract infection (20, 21). In addition, due to the decline in estrogen levels in menopausal women, the urethral mucosa may also undergo atrophic changes, which leads to a decrease in epithelial cell glycogen, which reduces the vaginal flora from glycogen-dependent lactic acid bacteria and increases Escherichia coli, leading to urinary tract infections (22).

Another systemic factor noted in our study to be closely related to both SIRS and urosepsis after PCNL is positive urine culture (OR = 3.21, *p* < 0.001; OR = 2.857, *p* = 0.01). Positive urine culture was previously considered to be a key factor associated with SIRS and urosepsis after PCNL surgery (23, 24). However, the mid-section urine culture cannot accurately reflect the microbial status of the upper urinary tract, especially some large or complex stones can cause urinary tract obstruction, and the urine produced by the affected kidney cannot reach the bladder (25). Emerging studies have shown that renal pelvic puncture urine culture and stone culture can predict uremia better than mid-bladder urine culture (26, 27). However, if the intraoperative puncture urine culture and stone culture results are selected to predict postoperative infection, greatly limit the value of their early predictions due to the long culture time. Therefore, preoperative urine culture is still the best predictor that can be obtained early.

The pre-operative blood routine indicators reflect the patient's basic state. Analyzing the results can conclude that the preoperative NLR level of SIRS (2.5 ± 1.0 vs. 2.0 ± 0.9, *p* < 0.001) and urosepsis (2.6 ± 1.1 vs. 2.0 ± 0.9, *p* < 0.001) patients is higher than that of the non-infected group. This is similar to the result that Sen believed, the incidence of sepsis in patients with NLR ≥ 2.5 was significantly higher than that of NLR < 2.50 (28). Bacterial infection can induce patient to release chemokines and inflammatory cytokines accumulate in the tissue microenvironment, stimulate chemotaxis and promote the production and release of a large number of neutrophils in the bone marrow, resulting in increased local and systemic neutrophil levels (29, 30). Hawkins has also reported that T lymphocytes and B lymphocytes are significantly reduced in both gram-positive and gram-negative bacteremia (31), which can lead to an increase in NLR levels. Because of its convenient and fast detection, NLR is expected to be an indicator for predicting SIRS and urosepsis.

The results of this study also showed that the incidence of SIRS after PCNL in patients with history of diabetes was 21.6% (21/97), which was significantly higher than that of patients without history of diabetes (11.5%, 76/661). Diabetes as an important factor in the high incidence of urinary tract infections has been noted (32). Long-term increasing serum glucose can cause the mobility, chemotaxis, phagocytosis, and adhesion of leukocytes, monocytes and macrophages decreasing, thereby reducing the body's ability to eliminate pathogens, and reducing organism immunity and resistivity (33). The study of Jia showed that patients with type II diabetes were accompanied by chronic low-grade inflammation, and the expression of peripheral T cell programmed death factor 1 (PD-1) increased, and PD-1 could inhibit the function and proliferation of T cells, which are similar to the performance of immune cells in patients with sepsis (34). These changes cause the body's response to invading pathogens to be low, and once bacteremia is present during PCNL surgery, it can lead to the occurrence of SIRS. In this study, the history of diabetes as a prognostic factor was significantly related to the occurrence of SIRS after PCNL (OR = 1.985, *p* = 0.035).

Our research has certain limitations. The operation time included in this study was the overall operation time from the start of anesthesia to the end of the operation. Since the retrieved medical records did not specify the specific time of the start of the puncture, we could not accurately collect the time from the establishment of the puncture channel to the end of the operation. Due to this reason, the operation time may be meaningless in this study and the recording of relevant medical records will be more specific and perfect in the follow-up work. This study is a retrospective study conducted in a single center, which may lead to potential selection bias. Although the model established in this study shows good fit and discrimination, it still lacks external verification, which is expected to be confirmed in an independent cohort.

## Conclusion

In this study, we found that female gender, higher preoperative NLR, higher S.T.O.N.E. score, and positive urine culture were the most significant predictors of SIRS and urosepsis. Diabetes history is the predictor of SIRS. These data will help identify high-risk individuals and facilitate early detection of SIRS and urosepsis.

## Data Availability Statement

The original contributions presented in the study are included in the article/Supplementary Material, further inquiries can be directed to the corresponding authors.

## Ethics Statement

The studies involving human participants were reviewed and approved by Ethics Committee of the First Affiliated Hospital of Sun Yat-sen University. Written informed consent for participation was not required for this study in accordance with the national legislation and the institutional requirements.

## Author Contributions

RW and JL: project development and manuscript revising. YT: project development, data collection, data analysis, and manuscript writing. CZ: data collection, data analysis, and manuscript writing. CM: project development and data collection. CG: data collection. All authors contributed to the article and approved the submitted version.

## Conflict of Interest

The authors declare that the research was conducted in the absence of any commercial or financial relationships that could be construed as a potential conflict of interest.

## Publisher's Note

All claims expressed in this article are solely those of the authors and do not necessarily represent those of their affiliated organizations, or those of the publisher, the editors and the reviewers. Any product that may be evaluated in this article, or claim that may be made by its manufacturer, is not guaranteed or endorsed by the publisher.
